# Combined space stressors induce independent behavioral deficits predicted by early peripheral blood monocytes

**DOI:** 10.1038/s41598-023-28508-0

**Published:** 2023-01-31

**Authors:** Kira D. A. Rienecker, Katherine Grue, Maria Serena Paladini, Elma S. Frias, Valentina Frattini, Mia C. Borlongan, Austin Chou, Abel Torres-Espin, Karen Krukowski, Adam R. Ferguson, Susanna Rosi

**Affiliations:** 1 Department of Physical Therapy and Rehabilitation Science, University of California at San Francisco, San Francisco, CA USA; 2 Brain and Spinal Injury Center, University of California at San Francisco, San Francisco, CA USA; 3 Department of Neurological Surgery, University of California at San Francisco, San Francisco, CA USA; 4 Weill Institute for Neuroscience, University of California at San Francisco, San Francisco, CA USA; 5 San Francisco Veterans Healthcare System, San Francisco, CA USA; 6 Kavli Institute of Fundamental Neuroscience, University of California at San Francisco, San Francisco, CA USA; 7 Present Address: Altos Labs, Redwood City, CA 94065 USA

**Keywords:** Immunology, Biomarkers, Neuroscience, Learning and memory, Social behaviour

## Abstract

Interplanetary space travel poses many hazards to the human body. To protect astronaut health and performance on critical missions, there is first a need to understand the effects of deep space hazards, including ionizing radiation, confinement, and altered gravity. Previous studies of rodents exposed to a single such stressor document significant deficits, but our study is the first to investigate possible cumulative and synergistic impacts of simultaneous ionizing radiation, confinement, and altered gravity on behavior and cognition. Our cohort was divided between 6-month-old female and male mice in group, social isolation, or hindlimb unloading housing, exposed to 0 or 50 cGy of 5 ion simplified simulated galactic cosmic radiation (GCRsim). We report interactions and independent effects of GCRsim exposure and housing conditions on behavioral and cognitive performance. Exposure to GCRsim drove changes in immune cell populations in peripheral blood collected early after irradiation, while housing conditions drove changes in blood collected at a later point. Female mice were largely resilient to deficits observed in male mice. Finally, we used principal component analysis to represent total deficits as principal component scores, which were predicted by general linear models using GCR exposure, housing condition, and early blood biomarkers.

## Introduction

The coming decade of deep space missions will incur prolonged and simultaneous hazards, including Galactic Cosmic Radiation (GCR), altered gravity, and social isolation. The impact of these hazards on behavioral function poses a risk to astronaut health and mission-critical decisions. Furthermore, we have little data on human responses to hazards of deep space. In low earth orbit (LEO), Earth’s magnetosphere largely protects astronauts from GCR, an ionizing radiation composed of protons, helium nuclei, and high energy nuclei of heavy elements^1,2^. Of the crews that have traveled beyond LEO, none included women; nevertheless, rodent models frequently reveal sex differences in susceptibility to the effects of ionizing radiation^3,4^. Of LEO missions, only a handful have exceeded a continuous year in space, approaching durations of social isolation and altered gravity comparable to a deep space mission^5^. Consequently, rodent models are essential for anticipating risk and developing countermeasures against the effects of deep space hazards. While rodent studies have provided important data about individual and paired space-related stressors, the combined effects of GCR, altered gravity, and isolation remain unknown.

Individual charged particles and mixed fields are used to model GCR, and biological responses vary based on dose, type of ionizing radiation, and time since irradiation. Deficits can be observed as early as 1 week, and as late as 1 year after irradiation^1,6^. Simplified 5-ion GCR simulation (GCRsim), developed at the NASA Space Radiation Laboratory, is a GCR simulation field consisting of protons at two energies (1000 MeV/n, 250 MeV/n) as well as ^28^Si (600 MeV/n), ^4^He (250 MeV/n), ^16^O (350 MeV/n), and ^56^Fe (600 MeV/n) particles^7^. A round trip to Mars is estimated to incur 40–50 cGy of GCR irradiation^1,2^. Rodent studies using a 50 cGy dose have shown an elevated percentage of peripheral blood monocytes in males, spatial memory deficits in males^4^, altered activity on the open field in females, novel object recognition deficits in females, and passive avoidance alterations in females and males^8^. Sex-specific responses are prevalent, with females sometimes proving more resilient to ionizing radiation, though not exempt from maladaptive effects^1,4,6,9^.

Social isolation-induced stress impacts behavior. Prolonged social isolation in adulthood enhances the motivational value and anticipatory behaviors of a social encounter^10^, increases social interactions^11^, and impairs social memory^12,13^. Adult isolation can impair novel object recognition, induce territorial behaviors, and alter anxiety-like and risk assessment behavior in the open field^14^. Sex differences are reported, particularly when isolation occurs during developmental periods of sex-specific social behaviors^15,16^.

Astronauts and mice in prolonged microgravity experience bone loss, muscle atrophy, immune dysregulation, and cephalic fluid shifts^17–20^. In rodent ground models, altered gravity achieved by hindlimb unloading (HU), which puts the animal in head-down tilt and prevents use of the hindlimb, leading to bone and muscle atrophy, and fluid shift toward the head^21^. The system employs a non-invasive attachment of the tail via orthopedic traction tape to a pulley system that elevates the hindlimbs while allowing the mouse to travel across the cage on a track^22^. Four weeks of hindlimb suspension increases the percentage of monocytes and macrophages and decreases the percentage of B lymphocytes and mature red cells in bone marrow of mice^23^.

Paired spaceflight stressors such as irradiation and hindlimb unloading have been extensively explored for their combined musculoskeletal effects^24–26^. In contrast, the combined effects of spaceflight stressors on behavior are under-characterized. This is particularly true for GCRsim exposure, which only recently became a NASA Space Radiation Laboratory (NSRL) capability^7^. Recent experiments suggest effects of HU and 5 ion GCRsim compete in male rats. Sham HU animals were impaired in spatial habituation learning, but GCRsim + HU rats were not^27^.

The combined effects of GCRsim, isolation, and altered gravity on behavior and cognitive functions are still unknown. In this experiment, we investigated the interaction of GCRsim and housing condition-group, social isolation (SI), and social isolation + hindlimb unloading (SI + HU)—in female and male mice irradiated at 6 months of age. We first characterized behavioral, cognitive, and sensorimotor function, and report interactions and independent effects of GCRsim and housing condition. Second, we measured alterations of immune populations in peripheral blood at early and late time points after irradiation. Third, we modeled the relationships between altered biomarkers in early peripheral blood and later behavioral deficits and tissue damage. We used dimension reduction techniques to generate principal component scores representing relationships between identified deficits and built ANCOVA models to determine if GCR exposure, housing condition, and early blood biomarkers predict these scores.

## Results

**Figure 1:**
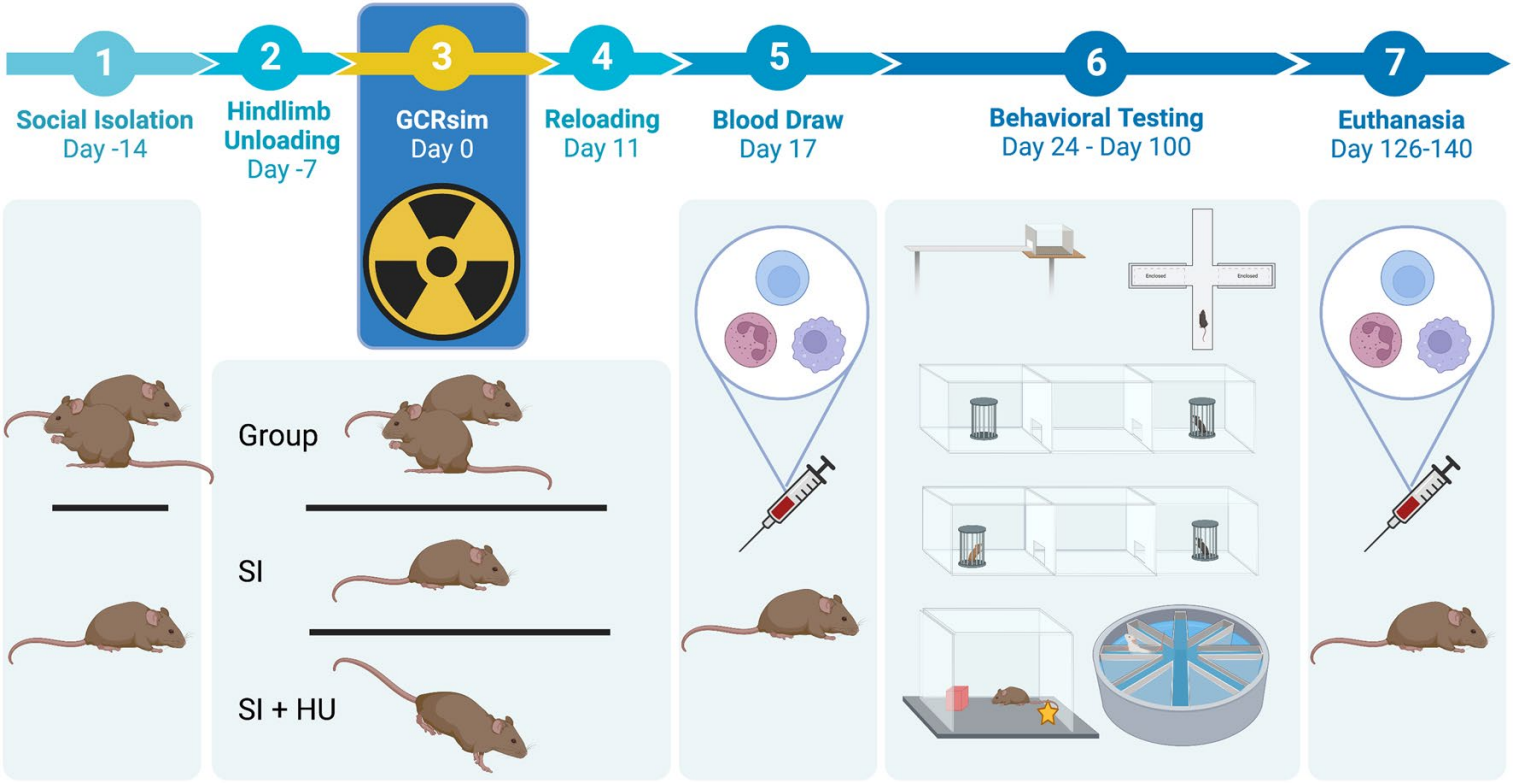
Experimental Design. Days are relative to GCRsim exposure, designated Day 0. On Day-14, animals were split into group housing and social isolation (SI) housing. On Day-7, socially isolated animals in the SI + HU group began hindlimb unloading (HU). GCRsim dose (0 cGy or 50 cGy) was delivered in one exposure on Day 0. All animals were loaded into the beam line housing array for dose delivery. SI + HU animals remained in hindlimb unloading posture. On Day 11, SI + HU animals were reloaded, and given a recovery period before being shipped from Brookhaven National Lab to UCSF. Blood was collected by tail vein on Day 17, and behavioral testing began with the Balance Beam on Day 24. Behavioral testing continued with EPM, Three Chamber Social Approach, Open Field, NOR, and RAWM, concluding around Day 100. Animals were euthanized around Day 126–140, and blood was collected by cardiac puncture for analysis. Created with BioRender.com.

All experiments were conducted in accordance with the National Institutes of Health Guide for Care and Use of Laboratory Animals and were approved by the Institutional Animal Care and Use Committee of University of California and Brookhaven National Laboratory. This study is reported in accordance with ARRIVE guidelines. The experimental design is outlined in Fig. 1.

### Effects of combined stressors and biological sex on spatial learning

**Figure 2:**
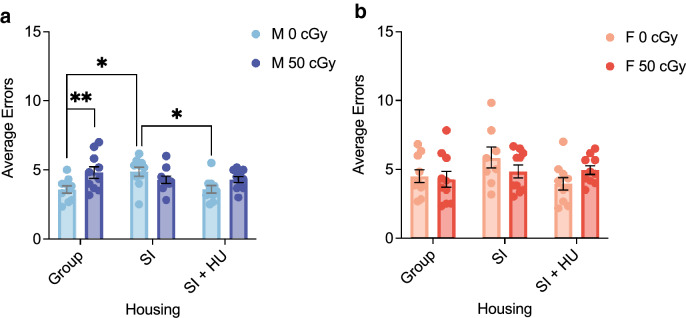
Combined stressors and biological sex differences in spatial learning. Radial Arm Water Maze (RAWM) was used to measure spatial learning under different housing and treatment conditions. Total Average Errors over 6 trials for Males (**a**) and Females (**b**). A two-way ANOVA showed a significant interaction between GCR and Housing for Total Average Errors (F(2,54) = 4.838, *p* = 0.012, η^2^ = 0.152). The simple main effect of GCR (F(1,54) = 8.340, *p* = 0.006, η^2^ = 0.134) showed a significant increase in average errors in 50 cGy compared to 0 cGy group housed males (mean diff 1.233, 95%CI(.377 to 2.090), *p* = 0.006), and the simple main effect of housing (F(2,54) = 6.036, *p* = 0.004, η^2^ = 0.183) showed a significant increase in average errors in 0 cGy SI males compared to 0 cGy group housed (mean diff 1.293, 95%CI(.238 to 2.349), *p* = 0.011) and SI + HU housed males (mean diff 1.277, 95%CI (.221 to 2.332), *p* = 0.013). Males: n = 10 per group for 60 total mice. Females: n = 10 for all groups except Group 50 cGy (n = 9), SI 0 cGy (n = 8) for 57 total mice. *p < 0.05, **p < 0.01.

The Radial Arm Water Maze (RAWM) assess spatial learning by counting the errors mice make before locating the escape platform inside one of the eight maze arms. As mice learn to navigate the maze, they reduce the number of errors. Here, we investigated combined effects of GCRsim exposure, social isolation, and hindlimb unloading on spatial learning using 6 trials on a single day of RAWM. Testing took place 13–14 weeks after HU mice were reloaded (Table 1). For male mice, there was an interaction between GCRsim and housing conditions (Fig. 2a). Exposure to GCRsim increased the average errors made by group housed males, supporting previous evidence that GCRsim exposure causes long term spatial learning deficits^4^. GCRsim exposure had no effect within SI and SI + HU groups. Housing condition showed simple main effects only among 0 cGy exposed mice. Mice in the 0 cGy social isolation (SI) housing condition made more errors compared to 0 cGy group and 0 cGy SI + HU conditions. Among 50 cGy exposed mice, housing had no effect on average errors.

**Table 1:** Animal ages and time elapsed during experiment.

Event	Age (Weeks)	Time from GCR (Weeks)	Time from reloading (Weeks)
Isolation start	25		
HU start	26		
GCR	27	0	
HU end at BNL (18 Days)	29	2	0
Blood draw at UCSF (7 days after reloading)	30	3	1
Balance beam (14 days after reloading)	31	4	2
EPM	33	6	4
Three chamber social approach	34–35	7–8	5–6
Adhesive removal	36–38	9–11	7–9
OF + NOR	39–41	12–14	10–12
RAWM	42–43	15–16	13–14
Euthanized	45–47	18–20	16–18

An increase in errors caused by SI or 50 cGy GCRsim exposure was not enhanced in the combination SI + 50 cGy group. The addition of HU reduced average errors compared to SI alone among 0 cGy mice. Errors made by male mice exposed to all three stressors together (SI + HU + GCRsim) were no different from any group. Our data suggest simultaneous SI and GCRsim exposure are not additive, and effects of HU and SI may compete. Female mice showed no deficits in spatial learning in any condition (Fig. 2b).

### Effects of combined stressors and biological sex differences on recognition memory

**Figure 3:**
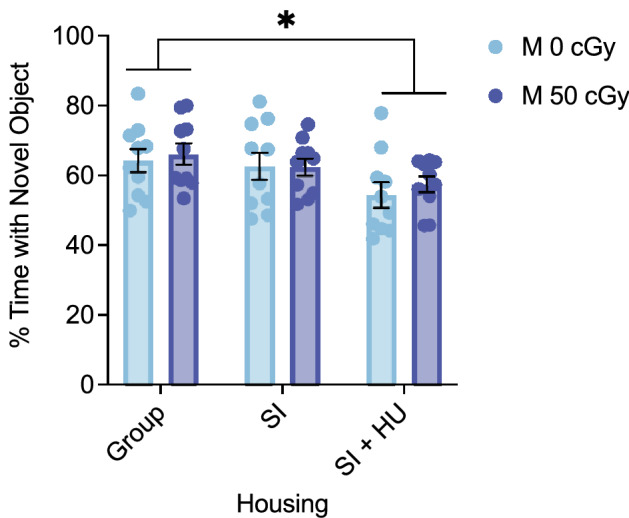
Combined stressors impair recognition memory. The novel object recognition (NOR) task was used to assess object recognition memory. (**a**) There was a significant main effect of Housing on % Time spent with the Novel Object during NOR Day 4 (F(2,54) = 4.523, *p* = 0.015, η^2^ = 0.143). Animals in the Social Isolation + Hindlimb unloaded condition spent a significantly lower % Time with the Novel Object compared to Group housed animals (mean diff 9.280, 95%CI (1.433 to 17.127), *p* = 0.015). Males: n = 10 for all groups. *p < 0.05.

Novel object recognition (NOR) measures object recognition memory, represented by the proportion of time mice spend investigating a novel object normalized to the total time spent exploring both the novel object and a familiar object. Mice able to adequately distinguish the novel and familiar object spend a greater proportion of their time with the novel object. In our test, the total percentage of time spent with the novel object was significantly lower for SI + HU male mice (GCR groups combined) than for group or SI housed mice (Fig. 3). These results show the paired stressors SI + HU impair object recognition memory. There was no significant effect of GCR in males, and there were overall no significant differences in NOR performance in female mice (Supplementary Fig. S1).

### Effects of combined stressors and biological sex on social exploration

**Figure 4:**
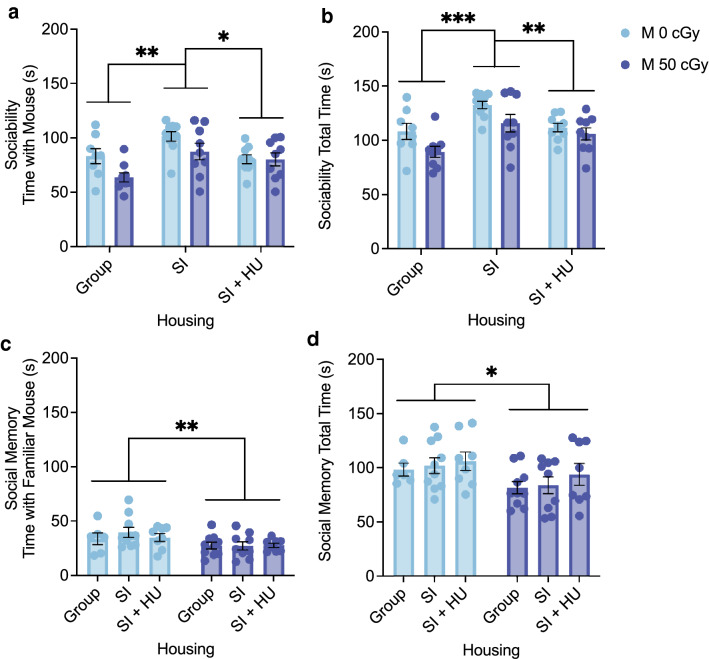
Combined stressors induce differences in social exploration. The three chamber social approach task was used to assess sociability and social memory. (**a**) The main effect of Housing on Time spent with the Mouse during the Sociability phase was F(2,48) = 7.312, p = 0.002, η^2^ =  0.234. SI mice spent significantly more time (s) with the social mouse than did Group (mean diff 20.966, 95%CI (7.005 to 34.926), *p* = 0.002) or SI + HU housed mice (mean diff 14.090, 95%CI (0.348 to 27.831), *p* = 0.043). (**b**) The main effect of Housing on Total Time spent with the Mouse + Cage was F(2,48) = 10.052, *p* < 0.001, η^2^ = 0.295. SI mice spent more total time than Group (mean diff 25.475, 95%CI(11.202 to 39.711), *p* < 0.001) or SI + HU housed mice (mean diff 15.444, 95%CI(1.412 to 29.476), *p* = 0.026). (**c**) The main effect of GCR on Time spent with the Familiar Mouse during the Social Memory phase was F(1,45) = 7.378, *p* = 0.009, η^2^ = 0.141. 50 cGy irradiated mice spent significantly less time with the Familiar mouse than did 0 cGy control mice (mean diff − 8.581, 95%CI (− 14.944 to − 2.218), *p* = 0.009). (**d**) The main effect of GCR on total time spent with mice was F(1,45) = 6.024, *p* = 0.018, η^2^ = 0.118. 50 cGy irradiated mice spent less total time than 0 cGy control mice (mean diff − 15.698, 95%CI (− 28.579 to − 2.816), *p* = 0.018). (SI = Social Isolation, HU = Hindlimb Unloading) Sociability Males: N = 9 for all groups except Group 0 cGy (n = 8) and SI 0 cGy (n = 10). Four animals were removed because due to errors in the task, and one because %Time with Mouse exceeded the 85% threshold. Social Memory Males: n = 10 for Group 50 cGy and SI 0 cGy, n = 9 for SI 50 cGy, n = 8 for SI + HU 0 cGy and SI + HU 50 cGy, and n = 6 for Group 0 cGy. Eight animals were removed due to errors in the task. One animal was removed due to %Time Novel Mouse exceeding the 85% threshold. *p < 0.05, **p < 0.01, ***p < 0.001.

The three-chamber social approach task measures sociability by quantifying the subject’s preference for investigating an age and sex matched mouse over investigating an empty cage. In male mice from SI housing (GCR groups combined) we measured an increase in time (s) spent with the mouse (Fig. 4a) and total time (s) spent with the mouse + cage (Fig. 4b) compared to group and SI + HU housed mice. Exposure to 50 cGy GCRsim (all housing conditions combined) reduced interaction time (Supplementary Figs. S2b,c). However, preference for the mouse over the cage (percent time spent with the mouse) was not changed under any stressor conditions (Supplementary Fig. S2a). These results suggest that social isolation alone increased, and GCRsim exposure decreased the time (s) a mouse spent engaging in social exploration behavior, but none of the stressors affected social preference for the mouse over the cage.

The three chamber social approach task next measures social memory by quantifying the subject’s preference for exploring an age and sex matched mouse they have never encountered before (novel) over a previously encountered one (familiar). Male mice exposed to 50 cGy GCRsim reduced time (s) they spent with the familiar mouse (Fig. 4c) and total time (s) spent with the familiar and novel mice (Fig. 4d), regardless of housing condition. However, the percentage of time spent with the novel mouse out of total time with both the novel and familiar mice was not affected by GCRsim or housing condition. Experimental mice adequately distinguished the novel and familiar mice, demonstrating social memory itself was not affected by the stressors (Supplementary Fig. S3a,b). The differences in raw time, but not normalized percentage, suggest differences in social exploration behavior, rather than social memory.

In female mice 50 cGy GCR exposure (all housing conditions combined) reduced total time with the mouse + cage in the sociability phase (Supplementary Fig. S2f). No other differences in sociability or social memory were measured (Supplementary Fig. S2d,e).

These results show within males, social isolation stress increased time spent with the mouse in the sociability phase, in agreement with previous reports that prolonged social isolation increases social interactions^11^. In contrast, GCRsim exposure reduced total performance on the tool in both the sociability and social memory phases. These differences are possibly due to changes in social exploration, rather than impairment of sociability and social memory. Finally, the response to GCRsim is dependent on the sex of the animals, where female mice show resilience to stressors that induce more extensive deficits in males.

### Combined space stressors effects on sensorimotor function and anxiety

Balance beam was used to measure sensorimotor function 14 days after the SI + HU animals were reloaded. Mice tested on their ability to cross a 5 mm wide beam of 80 cm in length towards a goal box. SI housed females traveled faster than controls on the 5 mm beam. There were no effects on average velocity for males (Supplementary Fig. S4). On the Elevated Plus Maze (EPM), mice displaying lower anxiety and higher risk-taking behaviors spend more time on the open arms and center of the maze. Female mice exposed to 50 cGy GCRsim spent less time on the open and center arms than controls (Supplementary Fig. S5b) Male mice showed no effects on the EPM (Supplementary Fig. S5a). In the Open Field (OF), mice displaying lower anxiety and higher risk-taking behaviors spend more time in the center zone of the arena. We measured no significant differences in time spent in the center zone of the Open Field for males or females (Supplementary Figs. S5c,d).

### Early effects of combined stressors on blood monocytes and natural killer cells

**Figure 5:**
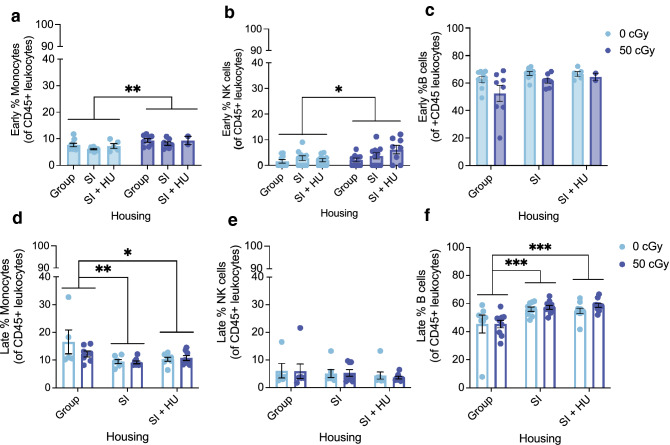
Combined stressors effects on early and late blood. Blood was collected from tail vein 3 weeks after GCRsim irradiation (**a, b, c**) and cardiac puncture at euthanasia (**d, e, f**). Percentages are of CD45 + cells. (**a**) Males exposed to 50 cGy had higher % monocytes than 0 cGy controls (F (1, 34) = 9.448, *p* = 0.004, η^2^ = 0.217) (mean diff 1.921, 95%CΙ (0.651 to 3.190), *p* = 0.004). (**b**) There was a main effect of GCRsim on % NK cells (F(1,50) = 4.756, *p* = 0.034, η^2^ = 0.087). Males exposed to 50 cGy had a higher % NK cells than controls (mean diff 1.801, 95%CI (0.142 to 3.459), *p* = 0.034). (**c**) % B cells trended toward a main effect of housing (F(1,34) = 3.179, *p* = 0.054, η^2^ = 0.158) and of GCRsim (F(1,34) = 3.474, *p* = 0.0709, η^2^ = 0.093). Males: n = 9 for Group 0 cGy, SI 0 cGy, n = 8 for Group 50 cGy, n = 7 for SI 50 cGy, n = 5 for SI + HU 0 cGy, n = 2 for SI + HU 50 cGy. (**d**) %Monocytes had a significant main effect of housing, F(2,37) = 6.382, *p* = 0.004, η^2^ = 0.256. Group housed mice had higher % Monocyte levels than SI (mean diff. 5.096%, 95%CI (1.411 to 8.780), p = 0.004) and SI + HU housed mice (mean diff. 3.882%, 95%CI (0.252 to 7.512), p = 0.033). (**e**) % NK cells for males. There were no main effects of housing or GCR. (**f**) For % B cells, there was a significant main effect of Housing. F(2,49) = 11.251, *p* < 0.0001, η^2^ = 0.315. Group housed mice had lower %B cells than SI (mean diff. − 11.203%, 95%CI (− 17.914 to -4.492), *p* < 0.001) and SI + HU housed mice (mean diff. − 11.287%, 95%CI (− 17.917 to − 4.656), *p* < 0.001). Males Monocytes and NK cells: n = 8 for SI + HU 0 and 50 cGy, SI 50 cGy, n = 7 for SI 0 cGy and group 50 cGy, n = 5 for group 0 cGy. Males B cells: n = 10 for SI + HU 0 and 50 cGy, SI 50 cGy, n = 9 for SI 0 cGy and group 50 cGy, n = 7 for group 0 cGy. *p < 0.05, **p < 0.01, ***p < 0.001.

We investigated peripheral blood at 17 days post-GCRsim exposure to determine if changes occurred in immune cell populations. In male mice, there was a significant main effect of GCRsim on the percent of monocytes (CD11b + of CD45 + cells) and natural killer cells (NK1.1 + of CD45 + cells) (Fig. 5a,b). Exposure to 50 cGy GCRsim increased the percentage of monocytes and NK out of total CD45 + cells in peripheral blood. There was no effect on B cells (Fig. 5c). Cell type percentages in female peripheral blood were unchanged (Supplementary Fig. S6).

### Blood monocytes predict the development of late behavioral deficits induced by combined stressors

Here, we investigate whether the percentage of blood monocytes measured 17 days after GCRsim exposure model correlate individually with late behavioral differences. We also compare the fit of a two-way ANOVA with GCRsim and housing condition modeling the deficit measure with the model fit of a two-way ANCOVA model adding the percentage of early blood monocytes as a covariate. As females were resilient to deficits, only male data was modeled.

RAWM average errors reflect spatial learning. Monocyte percentage and number of errors in the RAWM were not significantly correlated for males overall (r(38) = 0.188, *p* = 0.188), but individually were correlated for 0 cGy group housed (r(7) = 0.775, *p* = 0.014) data. Other groups considered individually did not have significant correlations. 50 cGy SI + HU animals were excluded from correlation because only data points for monocyte percentage survived quality control. The ANOVA containing GCRsim and housing condition modeling RAWM average errors in Fig. 2 had an overall model fit of F(5,54) = 3.474, *p* = 0.009, η^2^ = 0.243, adj R^2^ = 0.173. When monocyte percentage in early blood was included in the model as a covariate, the fit of the model improved (F(6,33) = 6.326, *p* < 0.001, η^2^ = 0.535, adj R^2^ = 0.450).

Time spent with the familiar mouse in the three chamber social interaction task reflected social exploration time, in the absence of discrimination impairment. Monocyte percentage was negatively correlated with time spent with the familiar mouse overall for males (r(33) = − 0.385, *p* = 0.022), but was not significant for any group considered individually. The fit of the two-way ANOVA containing GCRsim and housing condition was F(5,45) = 1.968, *p* = 0.102, η^2^ = 0.179, adj R^2^ = 0.088. The ANCOVA including monocyte percentage improved the fit (F(6,28) = 1.727, p = 0.151, η^2^ = 0.270, adj R^2^ = 0.114), though the model overall was still not significant, and there was no longer a significant effect of GCR (F(1, 28) = 1.291, *p* = 0.265, η^2^ = 0.044).

Other behavioral measures which showed deficits induced by GCRsim or housing condition (NOR impairment, Sociability Time with Mouse, Sociability Total Time, and Social Memory Total Time) were not predicted by Pearson correlation with the monocyte percentages. No behavioral deficits were significantly predicted by other blood cell types.

### Social Isolation is associated with reduced monocytes and increased B cells in late blood

We quantified CD45 + immune cells in blood obtained by cardiac puncture at the time of euthanasia to investigate changes at 18–20 weeks following GCRsim (Table 1). There was a main effect of housing condition on monocyte percentages in male mice. Group housed mice (0 and 50 cGy combined) showed a higher percentage of monocytes than SI and SI + HU conditions (Fig. 5d). Further, there was a main effect of housing on B cell percentages in male mice. SI or SI + HU housed mice had higher percentages of B cells than group housed mice (Fig. 5f). There were no changes in the percentage of NK cells (Fig. 5e), or any other cell populations investigated in male mice. There were no changes in cell populations in the blood of female mice (Supplementary Fig. S7).

### Predictive modeling of behavioral decrement and tissue health

To predict general long-term deficits identified in male mice using early blood biomarkers, we first used dimension reduction to describe relationships between individual deficits as principal component scores. We then compared ANOVA and ANCOVA models predicting the scores to determine whether including the percentage of monocytes measured early after GCRsim exposure improved the model fit.

We selected measures for the principal component analysis (PCA) which identified long-term deficits induced by GCR and housing conditions. Measures included sociability time with mouse, sociability total time, social memory time with familiar mouse, social memory total time, percent time with the novel object in the NOR task, RAWM total average errors, monocytes, and B cells percentages from cardiac blood. Sociability total time and sociability time with mouse were highly correlated (0.959), so sociability total time was discarded from the PCA to avoid collinearity.

**Figure 6:**
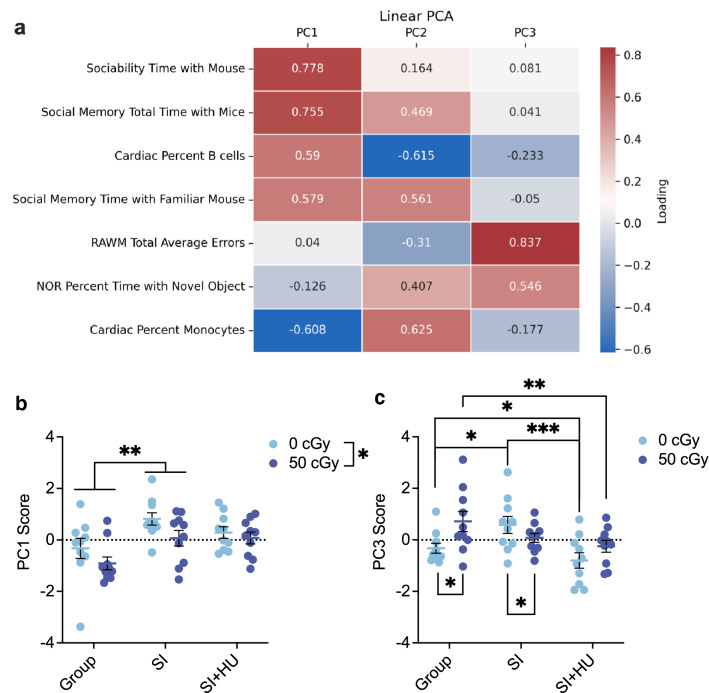
Linear PC Scores are modeled by Housing and GCRsim. (**a**) Loadings for features in the Linear PCA. Scores greater than |0.45| were used for interpretation of the PC. (**b**) PC1–Social exploration measures and B cells percentage from cardiac blood loaded strongly positively, while monocyte percentage from cardiac blood loaded strongly negatively on PC1. Two-way ANCOVA analysis had the best model fit for PC1. There was a significant main effect of housing (F(2,33) = (5.816, *p* = 0.007, η^2^ = 0.261) and GCRsim (F(1,33) = 6.694, *p* = 0.014, η^2^ = 0.169) on PC1. SI stress increased PC1 score compared to group housing (mean diff 1.155, 95%CΙ (0.294 to 2.015), *p* = 0.006). Sham irradiated animals scored higher on PC1 than 50 cGy exposed animals (mean diff 0.967, 95%CI (0.207 to 1.728), *p* = 0.014). (**c**) PC3- RAWM average errors and NOR percent time with the novel object loaded strongly positively. Two-way ANCOVA analysis including percent monocytes in early blood as a covariate had the best model fit for PC3. There was a significant interaction between GCRsim and housing condition (F(2,33) = 4.827, *p* = 0.014, η^2^ = 0.226). Compared to shams, the 50 cGy exposed mice scored higher in group housing (mean diff 0.906 (95%CI (0.015 to 1.797), *p* = 0.047) and lower in SI housing (mean diff − 0.948, 95%CI (− 1.890 to − 0.007), *p* = 0.048). Within all sham exposed animals, SI animals scored higher than both SI + HU (mean diff 2.496, 95%C (1.272 to 3.720), *p* < 0.001) and group housed animals (mean diff 1.276, 95%C (0.212 to 2.341), *p* = 0.014). Sham group housed males scored higher than SI + HU animals (mean diff 1.220, 95%C (0.019 to 2.420), *p* = 0.045). Within all 50 cGy animals, group housed scored higher than SI + HU animals (mean diff 2.180, 95%CΙ (0.482 to 3.878), *p* = 0.008). Males n = 9 0 cGy group, 0 cGy SI; n = 8 50 cGy group; n = 7 50 cGy SI; n = 5 0 cGy SI + HU; n = 2 50 cGy SI + HU. *p < 0.05 **p < 0.01, ***p < 0.001.

The linear PCA generated three PCs (Fig. 6a) with eigenvalues greater than 1 and explaining 70.462% of the variance together. Linear PC1 had an eigenvalue of 2.245 and explained 32.068% of the variance of the data. Social exploration measures and B cells percentage from cardiac blood loaded strongly positively while percent monocytes in cardiac blood loaded strongly negatively on PC1. Linear PC2 had an eigenvalue of 1.592 and explained an additional 22.748% of the variance in the data. Percent monocytes in cardiac blood and social exploration measures loaded strongly positively while B cells percentage from cardiac blood loaded strongly negatively on PC2. Linear PC3 had an eigenvalue of 1.095 and explained a further 15.645% of the variance in the data. RAWM average errors and NOR percent time with the novel object loaded strongly positively on PC3.

To assess whether non-linear associations among the outcomes or overfitting influenced PCA results, we benchmarked linear PCA results to a nonlinear PCA with bootstrapping limited to 3 PCs (Supplementary Table S1). Nonlinear PC1 had an eigenvalue of 2.472 and matched loadings with linear PC1. Nonlinear PC2 had an eigenvalue of 1.818 and loaded on the same components as linear PC3. Nonlinear PC3 had an eigenvalue of 1.704 and loaded positively onto B cell percentage in cardiac blood and negatively onto NOR percent time with the novel object. It did not closely match any linear PCs, suggesting nonlinear associations or overfitting may affect linear PC2. Overall, nonlinear PCA confirmed loadings for linear PC1 and linear PC3, so these were further investigated to determine if percent monocytes contributed predictive value to their models.

In the two-way ANOVA for PC1, the overall fit of the ANOVA was F(5,54) = 4.353, *p* = 0.002, η^2^ = 0.287, adj R^2^ = 0.221. When we included monocyte percentage in early blood as a covariate, the fit of the model improved (F(6,33) = 3.409, *p* = 0.010, η^2^ = 0.383, adj R^2^ = 0.270) (Fig. 6b). The Pearson correlation between monocyte percentage from early blood and PC1 score was not significant (r(38) = − 0.196, *p* = 0.226). Overall, GCRsim reduced while SI stress increased PC1 score. Monocyte percentage in early blood did not predict PC1 score on its own, but did it improve models as a covariate.

The overall fit of the two-way ANOVA was F(5,54) = 4.252, *p* = 0.002, η^2^ = 0.282, adj R^2^ = 0.216). Including percent monocytes in early blood as a covariate improved the fit of the model (F(6,33) = 6.925, *p* < 0.001, η^2^ = 0.557, adj R^2^ = 0.477) (Fig. 6c). Overall, the data show GCRsim exposure elevates PC3 scores among group housed mice and depressed them among SI mice. Among 0 cGy mice, SI stress elevated PC3 scores compared to group and SI + HU mice, while SI + HU stress also depressed scores compared to group housed. Among 50 cGy mice, SI + HU stress also depressed PC3 scores compared to group housed mice. There was no significant Pearson correlation between PC3 and monocyte percentage from early blood (r(38) = 0.177, *p* = 0.275). Monocyte percentage in early blood improved the fit of the model of PC3 scores but could not predict them on their own.

## Discussion

For the first time, we characterized the combined spaceflight stressors of GCRsim, social isolation, and hindlimb unloading on cognitive, behavioral, and blood cells measures in female and male mice. As previously reported, GCRsim exposure in group housed males increased errors in the RAWM^4^. Here, we showed housing conditions resulted in statistical differences in RAWM average errors among sham animals. Social isolation and hindlimb unloading have competing effects that last weeks after reloading. However, an experimental design using socially housed HU animals is needed to determine the independent effect of HU, as combined stressors can mask select responses to HU^28^. Finally, combinations of GCRsim with SI and SI + HU did not have additive effects on spatial learning.

We demonstrated combined social isolation and HU housing stressors impair object recognition memory, regardless of GCRsim exposure. Social isolation alone did not impair object recognition memory. Socially housed HU animals are required to determine whether this effect is due to HU alone or a combined effect of SI and HU. As in Krukowski 2021, GCRsim did not induce differences in object recognition memory^4^. While we show no NOR impairments in female mice, we note one other report using GCRsim showed females irradiated with 50 cGy had impaired object recognition^29^. In Raber 2020, B6D2F1 mice were comparable to our C57BL/6 mice in housing (group), age, and time since irradiation^8^. The discrepancy in results may be due to strain differences but may also result from testing methods. Mice in Raber 2020 were tested during the light period and the novel object arena used bright white lights^8^. Here, we tested mice during the dark (active) cycle and the novel object arena was lit under red lights. Testing during the animal’s rest phase (light period) and using bright light are both known to modulate performance during the object recognition task^30^. It is possible these factors also interact with GCRsim exposure during testing, resulting in group differences.

Social exploration during the three chamber social approach task was decreased by GCRsim exposure in male mice. Surprisingly, female mice exposed to GCRsim also showed a reduction in total time exploring the mouse and cage. Independently, housing conditions affected social exploration measures in males during this task. Social isolation housing in adulthood is expected to increase social interactions and the motivational value of social encounters^10,11^. Here, SI housing increased raw time spent with the mouse and total time exploring. As in the RAWM, the data suggested competing effects of SI and HU as SI + HU mice were not statistically different from group housed mice, even though SI + HU mice had been reloaded for weeks and both SI and SI + HU were still socially isolated. We cannot exclude that handling required to maintain animal health and safety during the hindlimb unloading period may have impacted sociability, particularly in males. However, while literature shows a lack of handling can obscure behavioral phenotypes^31^, there is little published about the effects of overhandling. In our study, there was no obvious grouping of sociability measures in males who required extra handling during the hindlimb unloading period. This suggests the results are not driven by the scores of a few highly handled animals. All mice were able to adequately discriminate between mouse and cage as well as novel and familiar mice. Together, the data show while sociability and social memory were intact, social exploration measures were increased by social isolation and decreased by 50 cGy GCR in male mice.

Our previous work with 5-ion GCRsim did not reveal differences in sociability or social memory, but 3-ion GCRsim and ^16^O exposure have altered performance in the three chamber social approach task^1,6^. Male mice exposed to 50 cGy of 3-ion GCRsim (protons, helium, oxygen) display less preference for a novel mouse over an empty cage compared to sham-irradiated mice^3^. This effect was not due to differences in total interaction time with the mouse and cage. Female mice showed no differences in measures of sociability. Males exposed to 50 cGy of 3-ion GCRsim also showed reduced preference for the novel mouse over the familiar mouse compared to the 15 cGy, but not the 0 cGy group. Again, there were no differences in total exploration time, nor were impairments in female cohorts observed^3^.

Male mice also showed deficits in the three chamber social task nine months after exposure to ^16^O. Both 0 and 40 cGy exposed males successfully differentiated mouse from cage and novel from familiar mice. However, discrimination of novel and familiar mice was reduced in the 25 cGy group, and total interaction time with conspecifics was reduced in the 40 cGy group compared to the 0 cGy^32^. Female mice also exhibited impaired discrimination between novel and familiar mice four months after exposure to 25 cGy ^16^O^33^. These data indicate 25 cGy ^16^O exposure impair social memory discrimination while 40 cGy impairs social exploration. Oxygen comprised only 6% of our 5-ion GCRsim, but the cumulative effect of 40–50 cGy of ^16^O or 5-ion GCRsim seems to reduce social exploration times while leaving discrimination intact.

We previously showed 50 cGy of 5-ion GCRsim results in increases in peripheral blood monocytes in males at 7 days after exposure^4^. Here, we demonstrated at 17 days post-exposure, GCRsim elevated peripheral blood monocytes and NK cells in males. In contrast, housing condition was the main driver of changes seen at 18–20 weeks following irradiation. Social isolation housing, regardless of hindlimb unloading status, reduced peripheral blood monocytes and increased B cells. These later effects may result from prolonged social isolation maintained throughout the experiment. No alterations were seen in our female animals, though social isolation may generate differences at earlier time points in female animals.

Overall, females were broadly resilient to deficits observed in male mice exposed to combined stressors. We previously reported female mice are resilient to deficits induced by GCRsim alone, and that this may be due to sex differences in microglial activation^9^. Male, but not female, mice exposed to 50 cGy of three ion GCRsim (proton, helium, and oxygen) showed extensive microglial activation in the dorsal hippocampus^3^. Furthermore, temporary microglial depletion rescued deficits induced in male mice by 5-ion GCRsim^4^. Conceivably, microglial activation in the hippocampus could contribute to deficits induced by combined stressors. Finally, we note deficits could be obscured by sex differences in task performance or the timelines of deficit progression.

We sought to use peripheral blood monocytes as a biomarker of male behavioral deficits. The percentage of peripheral blood monocytes measured at 17 days weeks post-GCRsim exposure correlated with RAWM average errors in 0 cGy group housed males and improved the fit of a general linear model of RAWM average errors. Monocytes were negatively correlated with raw time spent with the familiar mouse during the three chamber social approach task and improved the fit of the model containing GCRsim and housing condition. In conjunction with previous results^4^, we have demonstrated peripheral blood monocytes measured 1–3 weeks after GCRsim may be a useful predictor of individual behavioral decrements.

Further, blood monocytes collected early after GCRsim exposure improved models predicting relationships between deficit measures described by PCA. Linear PC1 represented positively loaded social exploration measures and B cell percentage and negatively loaded monocyte percentage from cardiac blood. Linear PC3 represented RAWM average errors and percent time with the novel object during NOR. These PC models describe unique variation in the relationship between deficits induced by space stressor, to which early blood monocyte percentage contributes, but cannot predict alone.

We do not know whether monocytes can be used to predict behavioral deficits before they manifest. This study does not reveal whether the reported behavioral deficits already exist at the time tail vein blood was sampled. Key behavioral tests performed earlier after GCRsim and reloading, or concurrently with blood sampling could reveal this. Describing the progression of deficits is important for defining windows of biomarker utility and countermeasure efficacy. Through these studies, we are better equipped to anticipate and mitigate the risks of deep space travel and empowered to go where no one has gone before.

## Materials and methods

### Animals

All experiments were conducted in accordance with the National Institutes of Health Guide for Care and Use of Laboratory Animals and were approved by the Institutional Animal Care and Use Committee of University of California (San Francisco, Protocol Number: AN181839) and Brookhaven National Laboratory (BNL, Upton, NY). Female (N = 60) and male (N = 60) C57BL6/J wildtype (WT) mice 24 weeks of age were purchased from Jackson Laboratory and directly delivered to BNL in grouped cages. Mouse ages were timed for comparability to previous experiments and to represent a middle-aged adult astronaut^4^. One female animal died before the experiment began. Each experimental group was assigned 10 mice, except normally loaded SI females, which was assigned 9 mice. A power calculation was not used to determine sample size; sample size was selected based norms for behavioral testing and maximum total colony capacity for the lab staff. Female and male mice were housed in separate rooms at BNL on a normal 12:12 light cycle. Animals acclimated at BNL for 1 week before animals in the two social isolation groups were separated into isolation housing in HU cages at 25 weeks of age. All isolated animals subsequently remained isolated for the duration of the experiment. Group housed controls remained in standard cages. Mice in the SI + HU group were unloaded at 26 weeks of age by a pulley apparatus with orthopedic traction tape as previously described^21,22,28^. The SI-only group remained normally loaded. Animals were checked twice a day, and re-loading events were recorded and corrected. At 27 weeks of age, animals underwent simplified 5-ion Galactic Cosmic Ray simulation (GCRsim) exposure at the NASA Space Radiation Laboratory during their light cycle (specifics described under ‘GCRsim’). Sham irradiated animals were loaded into the ‘mouse hotel’, but not placed on the beam line. The SI + HU group remained unloaded during irradiation. Animals were reloaded at 29 weeks of age, after a total of 18 days of HU. Following 5 days of recovery, mice were shipped to UCSF via World Courier™ for behavioral and cellular analysis. At UCSF, animals were housed on a reverse light cycle in environmentally controlled conditions (12:12 h light:dark cycle at 21 ± 1 °C; ~ 50% humidity) with access to food and water ad libidum. Female and male mice were housed in the same room. Animal ages, time elapsed since GCRsim, and time elapsed since reloading for all experimental assays are listed in Table 1.

### GCRsim

Mice were irradiated with 50 cGy of the NASA-approved simplified GCRsim protocol during the experimental campaign NSRL21A. The simplified GCRsim consisted of 35% protons (1000 meV), 1% silicon, 18% helium, 6% oxygen, 1% iron, and 39% protons (250 MeV). Energies were 1000 MeV/n protons (LET = 0.20 keV/µm), 600 MeV/n silicon ions (LET = 50.4 keV/µm), 250 MeV/n helium ions (LET = 1.60 keV/µm), 350 MeV/n oxygen ions (LET = 20.9 keV/µm), and 250 MeV/n protons (LET = 0.40 keV/µm). Mice were transported to the NSRL the night before exposure and returned to the animal care facility several hours after irradiation. During each exposure, mice were loaded into 7.3 × 4.0 × 4.0 cm polystyrene restraint boxes with air holes and mounted on the beam line. SI + HU animals remained at a declining angle by means of a wire attached to the tail harness and fixed to the restraint box holder outside the box. Due to the size of the box, hindlimbs could touch the wall of the restraint box. The beam spot size was 60 × 60 cm and animals were placed in the center of the field to assure best uniformity. The measured doses for the four runs were 50.0020 cGy delivered over 17.46 min, 50.0034 cGy delivered over 12.28 min, 50.0029 cGy delivered over 27.99 min, and 50.0021 cGy delivered over 42.76 min.

### Behavioral assays

Animals were handled to habituate to investigators and room settings for one week prior to the start of behavioral testing. Behavioral assays were performed in dark rooms during the animals’ wake cycle. All assays were recorded using an overhead camera connected to the Ethovision XT 12.0 tracking system (Noldus Information Technology). A combination of Ethovision software analysis and scoring manually by one investigator was used for analysis. Ethovision and investigator scores were compared for cross-validation, and a second investigator scored trials where Ethovision and the first investigator differed by more than 5% of the score. All behavioral analysis was performed by two female investigators. Blinding to the group condition was not possible, due to the isolation housing and limited personnel. Behavioral assessments took several weeks due to the size of the cohort. Trials were balanced so all experimental groups were represented on each day of an assessment. For some assessments, males and females were run on alternate days. Equipment was cleaned with 70% ethanol between all trials. The order of behavioral tests was: Balance Beam, Elevated Plus Maze, Three Chamber Social Approach Task, Adhesive Removal, Open Field, Novel Object Recognition, and Radial Arm Water Maze.

#### Balance beam

Fourteen days after reloading when the mice were 31 weeks of age, the balance beam was used to evaluate motor function^34,35^. The Balance Beam was manufactured by Conduct Science (ME-BB-M01) and consisted of a level plastic beam 80 cm long and of either 11 mm or 5 mm width, elevated 60 cm above the floor by two posts, with a dark goal box on the left, and a starting platform on the right. A hammock was placed below the beam to cushion falls. Two overhead lights were directed onto the starting platform and beam, while the goal box remained shaded. Testing was conducted in the housing room. Mice were habituated for 2 min inside the dark goal box and were allowed to freely explore the goal box and the 11 mm beam. Mice rested in the home cage for 10 min before the training phase. During the training phase, mice were placed in the center of the 80 cm long, 11 mm wide beam, and were allowed to move freely toward either end of the beam. Trials ended when the mouse entered the goal box, or when 2 min had elapsed. The mouse was allowed 15 s in the goal box upon arrival before it was removed back to the home cage and given a 10-min rest period before the Testing phase. During the Testing phase, the 11 mm beam was replaced with the 5 mm beam. The mouse was placed on the beam just past the right-hand starting platform, to encourage travel across the beam. The mouse was allowed to freely traverse the beam, and the trial ended when the mouse reached the goal box or when 2 min elapsed. Main measures of the assay were distance traveled and velocity. Footfalls were not quantified due to frequent use of a ‘scootching’ strategy, where the mouse pinched the sides of the beam between its hind paws to move forward.

#### Anxiety

Anxiety was assessed at 33 weeks of age using the Elevated Plus Maze (EPM) and at 39 weeks of age using the Open Field (OF)^4,36,37^. The EPM apparatus consisted of two exposed open arms (35 cm) opposite each other, orthogonal to two enclosed arms (30.5 cm) opposite from each other, creating a ‘plus’ shape with a center platform of 4.5 cm^2^. The apparatus was elevated 40 cm off the floor and bright white lights were used to illuminate the open arms. Individual mice were placed in the center zone, with their nose directed into an enclosed arm. Activity on the maze was recorded over 5 min. Time in the open arms + center was recorded in seconds. Animals that fell from the apparatus were replaced, but their data was excluded from analysis.

The Open Field test took place in a 30 cm by 30 cm arena for 10 min under red lighting. Mice explored freely and activity was recorded. Time spent (in seconds) in the center 17 cm by 17 cm zone was analyzed to assess anxiety-like behavior.

#### Sociability and social memory

The three-chamber social approach task was conducted when animals were 34–35 weeks of age to test sociability and social memory^4,38^. Individual animals were placed in the center chamber of a three chamber arena, where the total arena was 72 cm by 50 cm, and each chamber was 24 cm by 50 cm. All three stages of the task took place in a dark room under red light. The first stage, or habituation phase, consisted of 10 min of free exploration without any objects in the arena. The second phase measured sociability by placing an empty cage (10 cm in diameter) into one chamber of the arena, and an age- and sex-matched mouse (conspecific) not previously encountered into an identical cage in the opposite chamber of the arena. Cages were approximately 30 cm apart. Experimental animals explored the arena for 10 min. Interactions with the empty cage or conspecific mouse were defined as instances when the nose point of the experimental mouse was < 5 mm from the respective cage. Placement of the conspecific mouse was alternated between the left and right chamber for each trial. The first 5 min of exploration were used for analysis. Time (in seconds) with the cage and conspecific mouse were recorded for analysis. Sociability was assessed using % interaction time with mouse = ((time of nose interaction with mouse)/(total time of nose interaction with mouse + empty cage)) × 100. Social memory was measured in the third phase of the task. A novel sex- and age-matched mouse was placed into the previously empty cage. Exploration was measured for 5 min, and time (in seconds) interacting with the novel and familiar mouse already encountered in the previous phase was recorded. Social memory was assessed using the % interaction time with novel mouse = ((interaction time with novel mouse)/(total nose interaction with novel + familiar mice)) × 100. Conspecifics consisted of two cages (four mice per cage) of nonaggressive, sex- and age-matched nonlittermate mice). Investigators avoided odor mixing by cleaning the apparatus and cages with 70% ethanol and changing gloves each time they touched a new mouse. All four mice were used in all groups, and no aversions were noted.

#### Novel object recognition

The novel object recognition (NOR) assay was conducted when the animals were 39–41 weeks of age, to assess hippocampal-dependent recognition memory^4,39^. A 30 cm by 30 cm by 30 cm arena was used under red lighting. During the habituation phase on the first 2 days, animals explored freely for 10 min per day. During the training phase on day 3, two identical Duplo red blocks were secured to the floor of the arena in opposite corners. Mice were given 5 min to freely explore the objects, and interactions were defined as instances where the nose point was < 5 mm from the object. During the testing phase on day 4, one of the Duplo red blocks (familiar object) was replaced with a novel object–a Duplo orange flower. Mice were given 5 min to freely explore. Percent time with the novel object was defined as the time in seconds spent with the novel object divided by the total time in seconds spent with the novel + familiar objects.

#### Radial arm water maze (RAWM)

The radial arm water maze was conducted to test spatial learning when the animals were 42–43 weeks of age^4,40^. The maze was a circular pool with a diameter of 118.5 cm and eight arms 41 cm in length. The pool was filled with water and a non-toxic white paint (Crayola, 54-2128-053) was added to make the water opaque. An escape platform was placed at the end of one of the arms, hidden from view below the surface of the water. Visual cues were placed around the room. Animals ran 1 day of 6 consecutive 60 s trials with a 10 min interval. At the start of each trial, animals were placed in a different arm not containing the escape platform. Animals unable to locate the platform were guided to the platform. Once any animal reached the platform, they remained on the platform for 10 s and were returned to the holding cage. The number of errors (entries into an arm without the escape platform) was determined by Ethovision software. The number of errors in trials 1–6 were averaged to calculate the “day average”.

### Tissue collection

Upon arrival at UCSF, seven days after reloading (see Table 1), blood was collected via tail vein puncture. A small nick was made in the tail vein using a scalpel and ~ 40 µl of blood was removed using a pipette and placed in a small tube containing EDTA (Sigma). Blood was used to measure early changes in peripheral blood biomarkers.

Following the completion of behavioral assays, animals were lethally overdosed using carbon dioxide. Once completely anesthetized, the chest cavity was opened, and mice were perfused with 1 × phosphate buffer solution, pH 7.4 (Gibco) until the liver was clear (~ 1–2 min). Following PBS perfusion, the whole brain was quickly removed, and one hemisphere was either immediately processed for microglia counts by flow cytometry or drop-fixed in 4% PFA for immunohistochemistry. The other hemisphere was dissected by brain region and snap frozen on dry ice and stored at −80 °C.

### Flow cytometry

#### Blood

Circulating leukocyte populations were evaluated 17 and 100 days following GCRsim. Blood was stained with surface antibodies for 30–60 min at room temperature^41^. The antibody panel included anti-CD45 [fluorescein isothiocyanate (FITC)–conjugated; BD Biosciences], CD11b [PacificBlue–conjugated; BD Biosciences], CD3 [allophycocyanin (APC)–conjugated; BD Biosciences], CD4 [AmCyan–conjugated; BD Biosciences], CD8 [PerCp-Cyanine5.5–conjugated; BD Biosciences], CD19 [APC-Cy7–conjugated; BD Biosciences], Ly6G [phycoerythrin cyanine-7 (PE)–conjugated; BD Biosciences], and NK1.1 [PE–conjugated; BD Biosciences]. Subpopulations were identified as described in Supplementary Table S2. Data were collected on Canto II (BD Biosciences) and analyzed using FlowJo software (v10, Tree Star Inc.). Markers for immune cell populations are listed in Supplementary Table S2.

### Data analysis

Statistical analyses of individual assays were performed using Graphpad Prism 9. Female and male cohorts were analyzed separately. Two-way ANOVAs were used to investigate the interaction between GCR (0 vs. 50 cGy) and housing condition (group, social isolation, and social isolation + hindlimb unloading). Bonferroni corrections were applied for pairwise comparisons. Individual statistical analysis is denoted in the figure legends. Data are expressed as the mean ± standard error of the mean unless otherwise stated. Mouse 373 (Female) was removed from analysis because of an underdeveloped eye which may have impaired performance of the behavioral tasks.

#### ANCOVA and pearson correlations

ANCOVA was used to predict continuous behavioral variables based on the interaction of GCR exposure with housing condition and a covariate of percent monocytes in early blood. Analysis was performed with IBM SPSS Statistics V.27. The continuous percent monocytes variable was mean-centered to reduce collinearity and GCR and Housing were dummy-coded. Pearson correlations were carried out to investigate the correlation of individual measures.

#### Principal component analysis

PCA was performed using IBM SPSS Statistics V.27. Measures were selected for the PCA if they displayed deficits due to GCRsim or housing. Potential PC attractors and sources of duplicate information were eliminated. Little’s test was performed to test the null hypothesis that the data was missing completely at random. Little’s test failed to reject the null hypothesis in all analyses, and mean substitution was used to replace missing values (10.95% of the dataset was missing). Linear PCA was performed without rotation and iterated 25 times. PCs were retained using the eigenvalue > 1 rule (Kaiser criterion) and the Cattell’s elbow rule (components above the elbow of the scree eigenvalue plot). PCs were interpreted and named based on loadings >|0.45|. PC scores were calculated for each subject using the regression method (range − 1.0 to 1.0) on each of the retained PCs. We performed non-linear PCA with mode substitution and bootstrapping (balanced, 1000 samples, Procrustes rotation) to compare nonlinear PCs to the linear PCs. Only male data (N = 60) was analyzed, as females were largely resilient to deficits induced by GCRsim or housing condition.

## Supplementary Information

Supplementary Information.

## Data Availability

All data needed to evaluate the conclusions in the paper are present in the paper and/or the Supplementary Materials.
