# Supplementary material for: Combined space stressors induce independent behavioral deficits predicted by early peripheral blood monocytes
Source: Sci Rep. 2023 Jan 31;13:1749. doi: 10.1038/s41598-023-28508-0 (PMC9889764; doi:10.1038/s41598-023-28508-0)
Supplement: Supplementary file 1 — Supplementary Information. [file 41598_2023_28508_MOESM1_ESM.pdf]

Combined space stressors induce independent behavioral deficits predicted by early peripheral blood monocytes

Kira D. A. Rienecker[a,b], Katherine Grue[a,b], Maria Serena Paladini[a,b], Elma S. Frias[a,b], Valentina Frattini[a,b], Mia C. Borlongan[a,b], Austin Chou[a,b], Abel Torres-Espin[b,c,d], Karen Krukowski[a,b], Adam R. Ferguson[b,c,d,e], Susanna Rosi[a,b,c,d,f,g*]

Supplementary Figure S1 NOR Day 4

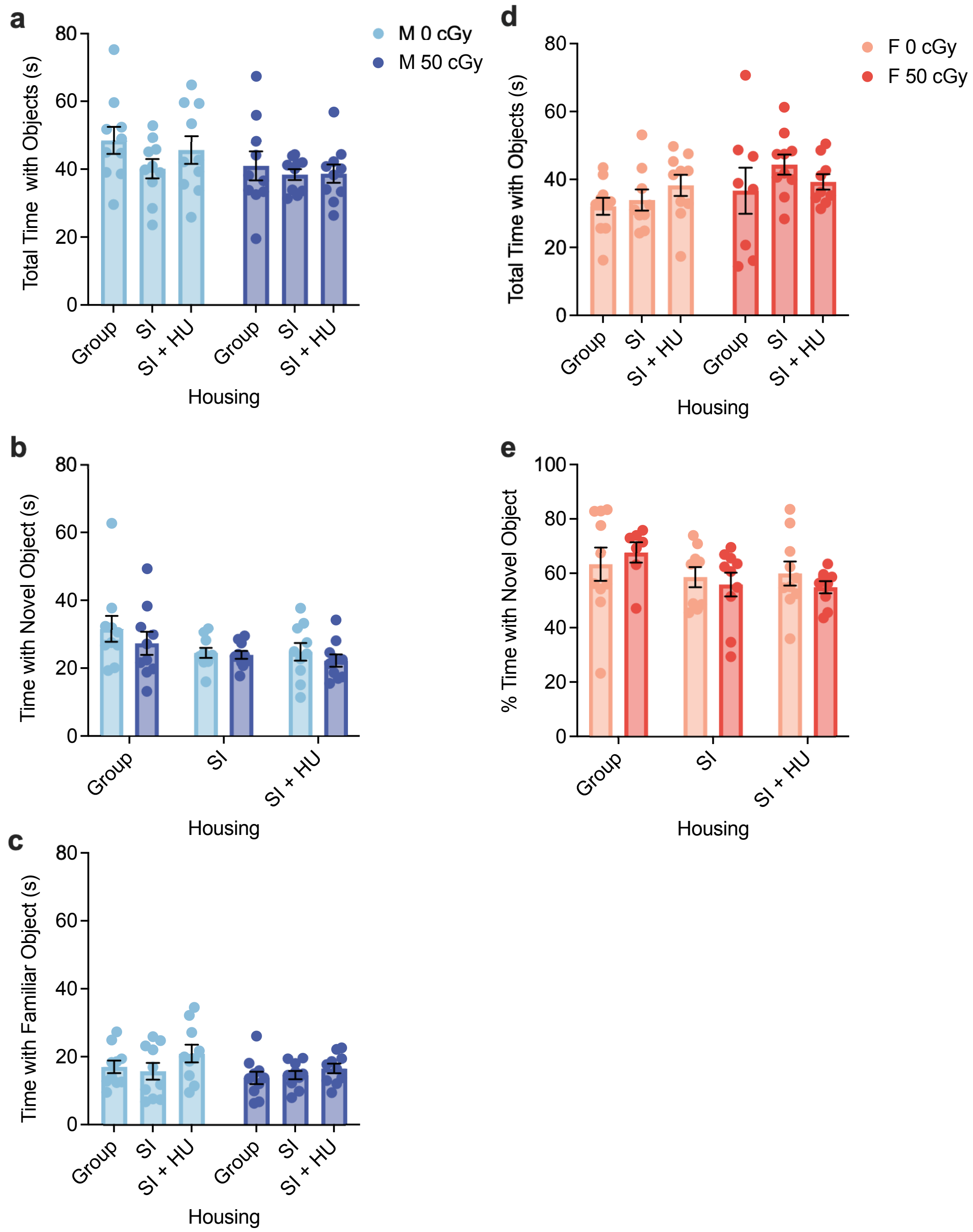

Supplementary Figure S2 GCRsim and sex differences in sociability

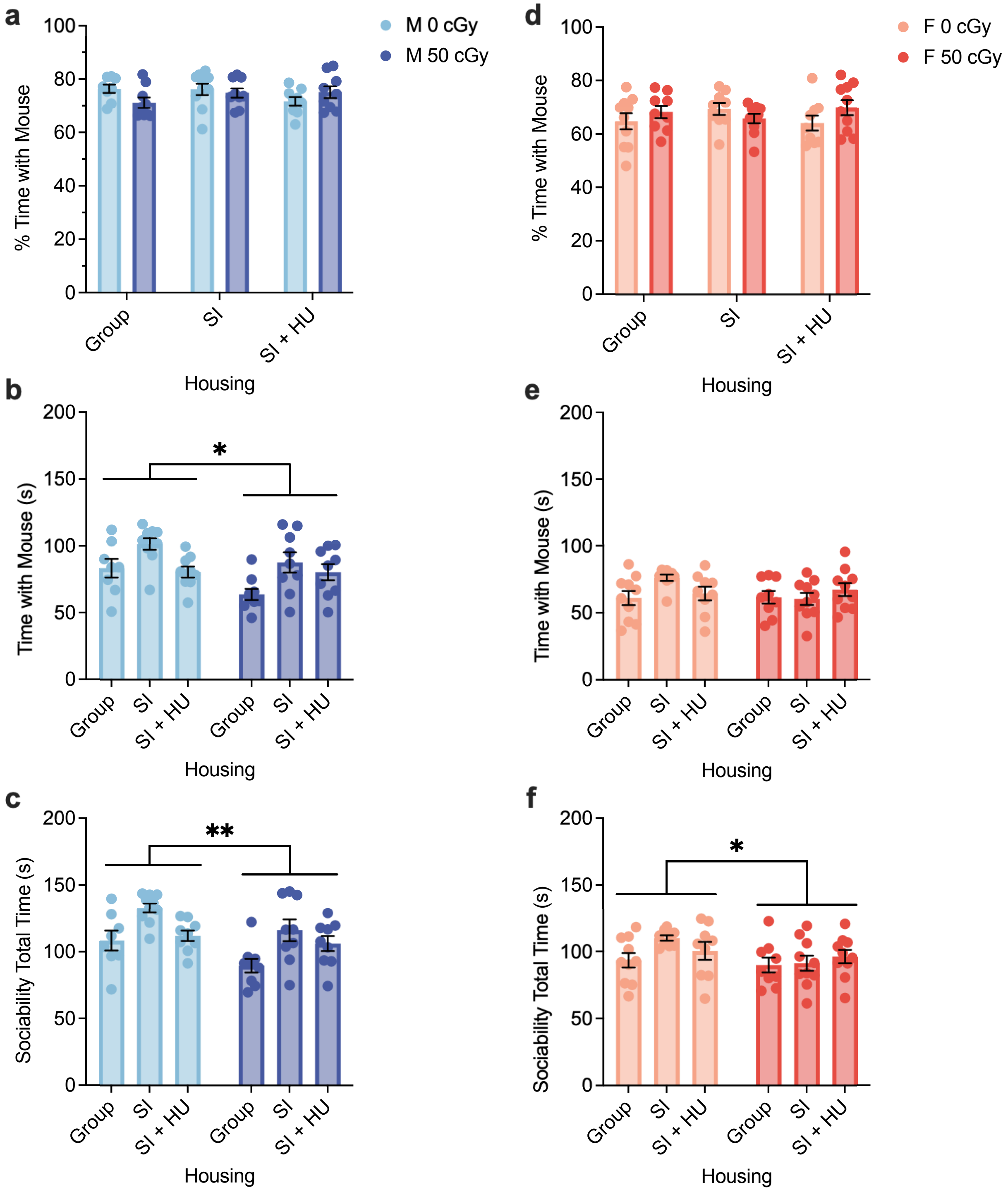

Supplementary Figure S3 Social Memory

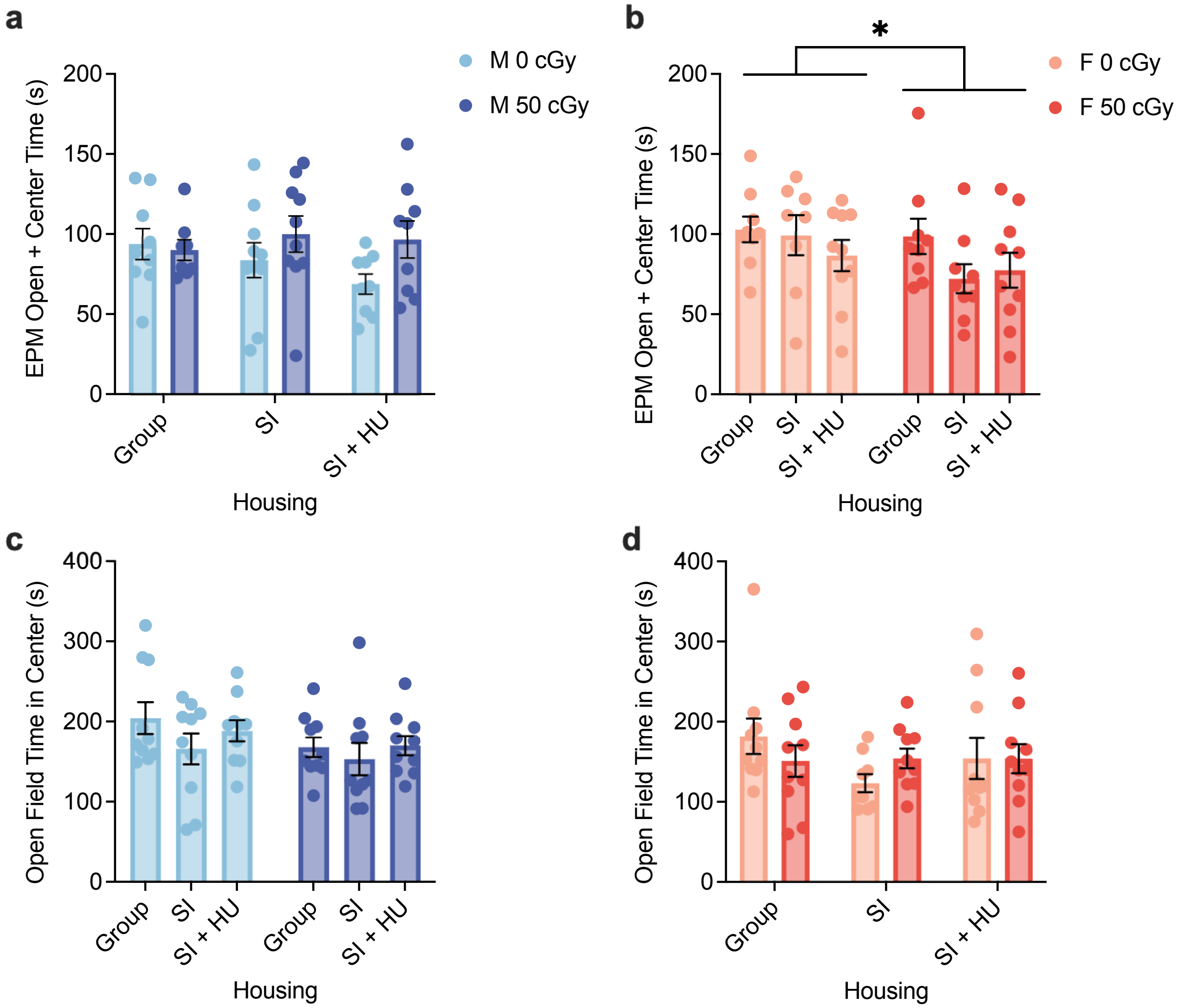

Supplementary Figure S4 Combined Stressors effects on sensorimotor function

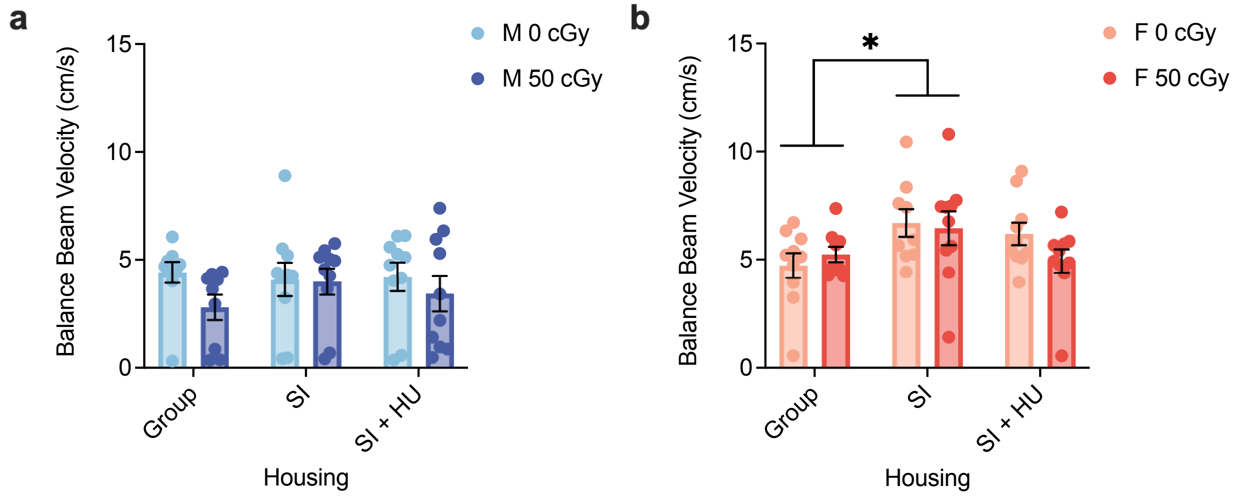

Supplementary Figure S5 Combined stressors effects on anxiety

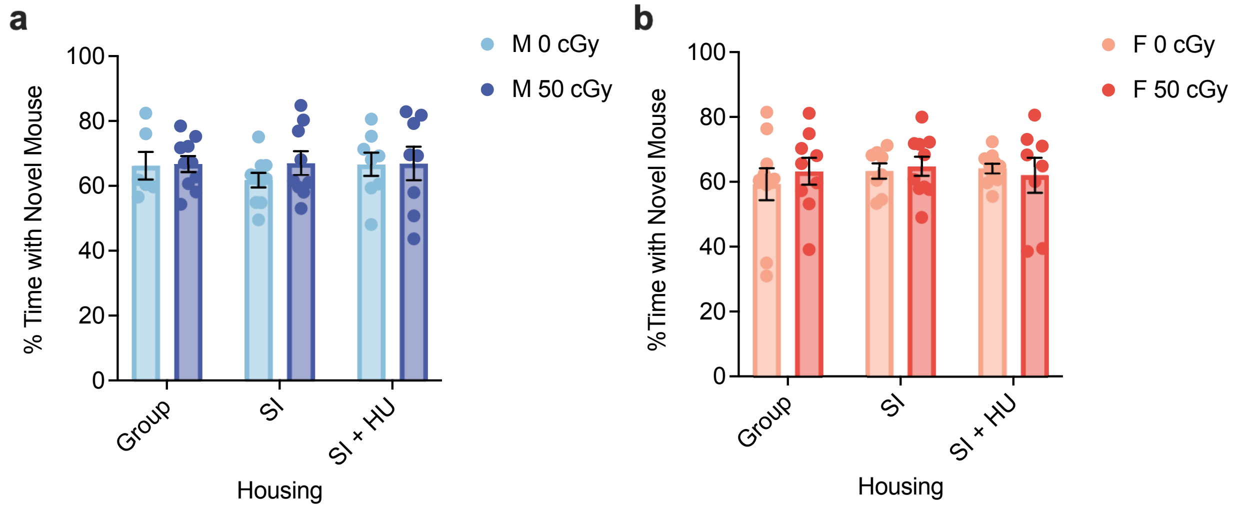

Supplementary Figure S6 Combined stressors do not alter early blood cell populations in females

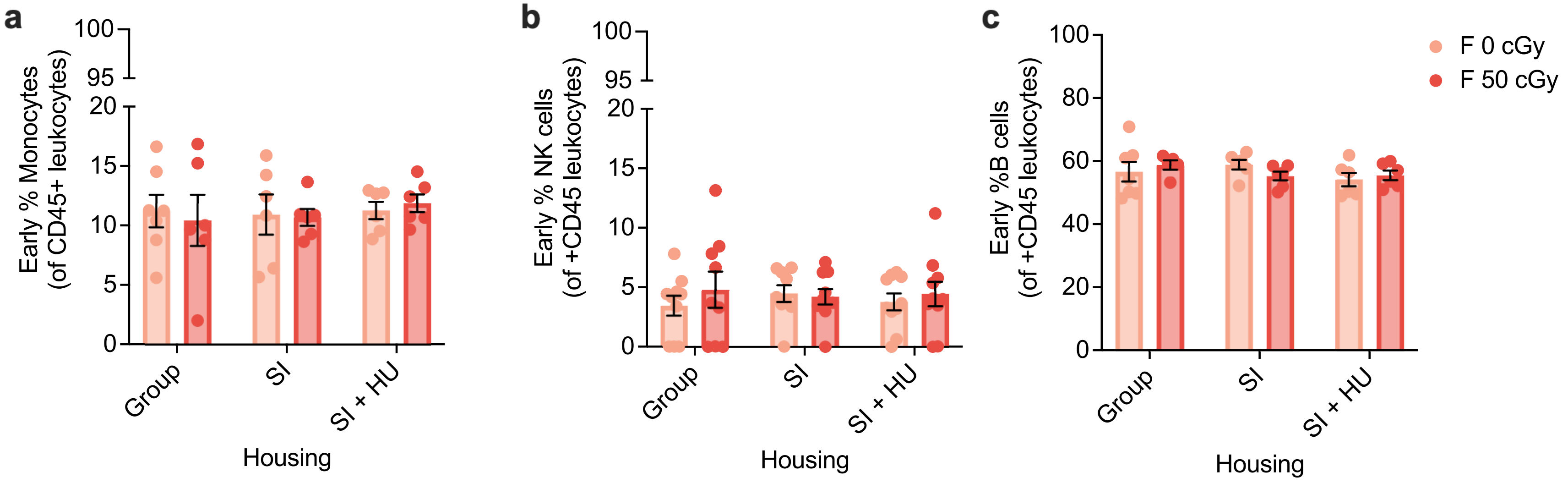

Supplementary Figure S7 Combined stressors do not alter late blood cell populations in females

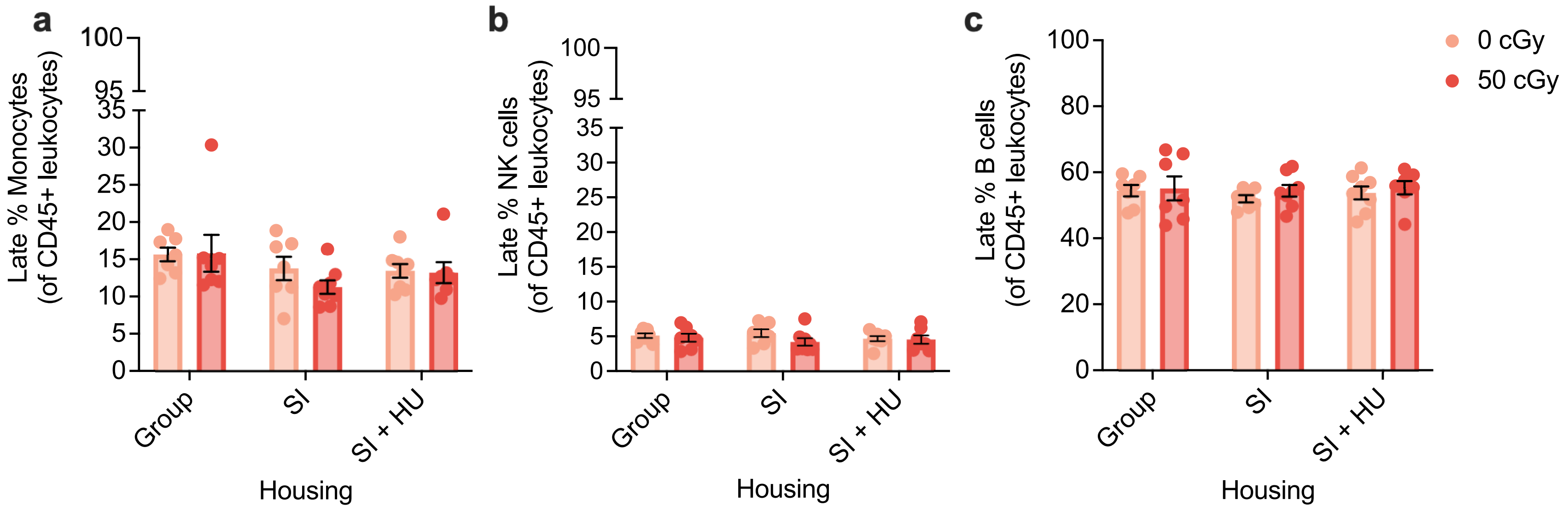

Supplementary Table S1 Semi-Supervised Nonlinear PCA

Component Loadings	PC1		95% Bootstrap CI		PC2		95% Bootstrap CI		PC3		95% Bootstrap CI	
	Loading	Bootstrap Mean	Lower	Upper	Loading	Bootstrap Mean	Lower	Upper	Loading	Bootstrap Mean	Lower	Upper
Sociability Time with Mouse	0.857	0.834	0.617	0.953	0.063	0.038	-0.516	0.36	-0.231	-0.184	-0.508	0.533
Social Memory Total Time with Mice	0.796	0.788	0.667	0.893	-0.154	-0.18	-0.395	0.075	-0.418	-0.423	-0.582	-0.215
Cardiac Percent B Cells	0.64	0.578	0.039	0.809	0.184	0.141	-0.427	0.627	0.657	0.707	0.343	1.012
Social Memory Time with Familiar Mouse	0.629	0.613	0.331	0.781	-0.482	-0.485	-0.898	-0.005	-0.398	-0.401	-0.698	-0.008
RAWM Total Average Errors	0.052	-0.066	-0.586	0.424	0.903	0.804	0.396	1.007	-0.161	-0.162	-0.744	0.51
NOR Percent Time with Novel Object	-0.178	-0.229	-0.561	0.095	0.584	0.499	-0.495	0.817	-0.82	-0.769	-1.081	-0.268
Cardiac Percent Monocytes	-0.515	-0.535	-0.714	-0.269	-0.606	-0.582	-0.9	-0.107	-0.433	-0.38	-0.769	0.268

Supplementary Table S2 Blood cell markers for flow cytometry

Immune Cell Population	Surface Staining
$CD8^+$ Cytotoxic T cells	**$CD45^+$, $CD3^+$, $CD8^+$**, $CD4^-$, $CD11b^-$, $NK1.1^-$, $CD19^-$, $Ly6G^-$
$CD4^+$ T helper cells	**$CD45^+$, $CD3^+$, $CD4^+$**, $CD8^-$, $CD11b^-$, $NK1.1^-$, $CD19^-$, $Ly6G^-$
B cells	**$CD45^+$, $CD19^+$**, $CD3^-$, $CD4^-$, $CD8^-$, $CD11b^-$, $NK1.1^-$, $Ly6G^-$
NK Cells	**$CD45^+$, $NK1.1^+$**, $CD3^-$, $CD4^-$, $CD8^-$, $CD11b^-$, $CD19^-$, $Ly6G^-$
Monocytes	**$CD45^+$, $CD11b^+$**, $CD3^-$, $CD4^-$, $CD8^-$, $NK1.1^-$, $CD19^-$, $Ly6G^-$
Neutrophils	**$CD45^+$, $Ly6G^+$**, $CD3^-$, $CD4^-$, $CD8^-$, $CD11b^-$, $NK1.1^-$, $CD19^-$

Supplementary Figure Legends

Figure S1 NOR Day 4. (**a**) Total Time with Objects for Males trended toward a significant main effect of GCR (F(1,54) = 3.805, p=0.0563, $\eta^2 = 0.066$). (**b**) Time with Novel Object for Males trended toward a significant effect of housing (F(2,54) = 3.159, p = 0.050, $\eta^2 = 0.105$) (**c**) Time with Familiar Object for Males trended toward a significant main effect of GCR (F(1,54) = 3.301, p=0.0748, $\eta^2 = 0.058$). (**d**) Total Time with Objects for Females trended toward a significant main effect of GCR (F(1,49) = 3.188, p = 0.080, $\eta^2 = 0.061$). (E) % Time with Novel for Females showed no significant main effects or interaction between GCR and Housing. Three female 50 cGy animals were excluded for failing to perform the task. Males: n=10 for all groups. Females: n = 9 for SI 0 cGy and SI+HU 50 cGy and n = 8 for group 50 cGy, n=10 for all others.

Figure S2 GCRsim and sex differences in sociability. The three chamber social approach task was used to assess sociability. Males (**a,b,c**) and Females (**d,e,f**). Both males and females showed adequate preference for the mouse over the cage (**a,d**). (B) Male mice receiving 50 cGy of GCRsim spent significantly less time with the mouse (F(1,48)=5.921, p=0.019, $\eta^2 = 0.110$) (mean diff -11.169, 95%CI(-20.398 to -1.940), p = 0.019) and (C) less total time with the cage and mouse together (F(1,48)=8.666, p=0.005, $\eta^2 = 0.153$) (mean diff -13.797, 95%CI(-23.220 to -4.373), p = 0.005). Female mice receiving 50 cGy of GCRsim also spent significantly less total time with the cage and mouse together (F(1,51)=4.262, p=0.044, $\eta^2 = 0.077$) (F), but showed no differences in time spent with the mouse (E). Sociability Males: n = 9 for all groups except Group 0 cGy (n=8) and SI 0 cGy (n=10). Four animals were removed because due to errors in the task. One male was removed because %Time with Mouse exceeded the 85% threshold. Females: n = 9 for SI 0 cGy, SI+HU 0cGy, SI+HU 50 cGy, n= 10 for all others. One female was removed for failing to perform the task. *p<0.05, **p<0.01

Figure S3 Social Memory. Percent Time spent with Novel Mouse for (**a**) Males and (**b**) Females. There were no significant interactions or main effects within either sex when examined by two-way ANOVA. Males: n=6 for group 0 cGy, n=7 for SI+HU 0 cGy, n=8 for SI+HU 50 cGy, n=9 for SI 50 cGy, n=10 for all others. Eight animals were removed due to errors in the task. One animal was removed due to %Time Novel Mouse exceeding the 85% threshold. Females: n=8 for SI 0 cGy and SI+HU 50 cGy, n= 9 for group 50 cGy, n=10 for all others. One animal was removed due to errors in the task, and two animals were removed due to %Time Novel Mouse exceeding the 85% threshold.

Figure S4 Combined Stressors effects on sensorimotor function. Balance beam was used to assess sensorimotor function 14 days after SI+HU animals were reloaded. (**a**) Males and (**b**) Females. There was a significant main effect of housing for female mice on the balance beam (F(2,52) = 3.702, p = 0.031, $\eta^2 = 0.125$). SI females traveled faster than group housed females (mean diff 1.594 cm/s, 95%CI(0.127 to 3.061), p = 0.029). Males: n=9 for group 50 cGy and n=10 for all others. Females: n=9 for SI 0 cGy and group 50 cGy, and n = 10 for all others.

Figure S5 Combined stressors effects on anxiety. Elevated Plus Maze (EPM) and Open Field (OF) were used to assess anxiety at different time points after exposure to GCRsim. Animals were 33 weeks of age during EPM testing and 39-41 weeks of age during OF testing. (**a**) EPM Open Arm + Center Time in 5 min for Males showed no differences. (**b**) There was a main effect of GCRsim on EPM Open Arm + Center Time for females, $F(1,48) = 4.417$, $p = 0.041$, $\eta^2 = 0.152$ (mean diff -16.765 s, 95%CI(-32.803 to -0.726), $p = 0.041$). Males: n= 10 for SI 0 cGy and SI 50 cGy, n=8 forgroup 50 cGy, n =9 for all others; Females: n=10 for SI+HU 0 and 50 cGy, n=9 for SI 0cGy and Group 0 and 50 cGy, n=8 for SI 50 cGy. Open Field (OF) Time in the Center of the arena in 10 min for (**c**) Males and (**d**) Females. Two way ANOVA within each sex for each task showed no significant effects. Males: n=10 for all groups. Females: n=10 for all groups except SI 0cGy and group 50 cGy, n=9. $^*p < 0.05$

Figure S6 Combined stressors do not alter early blood cell populations in females. Blood from female mice was collected from tail vein 3 weeks after GCRsim irradiation and analyzed using flow cytometry. All cells are % of CD45+ cells. (**a**) % Monocytes (**b**) % NK cells (**c**) % B cells. Females monocytes: n= 7 group 0 cGy, n=6 for all other groups. Female NK cells: n=9 for SI 0 cGy and group 50 cGy, n=10 for all others. Females B cells: n=7 group 0 cGy, n=5 group 50 cGy, n=6 for all other groups.

Figure S7 Combined stressors do not alter late blood cell populations in females. Blood from female mice was collected from cardiac puncture at euthanasia and analyzed using flow cytometry. All cells are percentages of CD45+ cells. (**a**) % Monocytes. (**b**) % NK cells. (**c**) % B cells. Females of all cell types: n=7 group 0 cGy, SI0 cGy, group 50 cGy; n=8 for all others.
